# Current Technologies and Future Perspectives in Immunotherapy towards a Clinical Oncology Approach

**DOI:** 10.3390/biomedicines12010217

**Published:** 2024-01-18

**Authors:** Subhamay Adhikary, Surajit Pathak, Vignesh Palani, Ahmet Acar, Antara Banerjee, Nader I. Al-Dewik, Musthafa Mohamed Essa, Sawsan G. A. A. Mohammed, M. Walid Qoronfleh

**Affiliations:** 1 Medical Biotechnology, Faculty of Allied Health Sciences, Chettinad Academy of Research and Education (CARE), Chettinad Hospital and Research Institute (CHRI), Chennai 603103, India; 2 Faculty of Medicine, Chettinad Hospital and Research Institute (CHRI), Chennai 603103, India; 3 Department of Biological Sciences, Middle East Technical University, 06800 Ankara, Türkiye; acara@metu.edu.tr; 4 Department of Pediatrics, Women’s Wellness and Research Center, Hamad Medical Corporation, Doha 00974, Qatar; nader.al-dewik@kingston.ac.uk; 5 College of Agricultural and Marine Sciences, Sultan Qaboos University, Muscat 123, Oman; 6 QU Health, College of Medicine, Qatar University, Doha 00974, Qatar; sawsan@qu.edu.qa; 7 Research & Policy Division, Q3 Research Institute (QRI), Ypsilanti, MI 48917, USA

**Keywords:** cancer, immunogenicity, gene therapy, immunotherapy, immune checkpoint, CAR T cell therapy, dendritic cell-based therapy, NK cell-based therapy, CRISPR-Cas9

## Abstract

Immunotherapy is now established as a potent therapeutic paradigm engendering antitumor immune response against a wide range of malignancies and other diseases by modulating the immune system either through the stimulation or suppression of immune components such as CD4^+^ T cells, CD8^+^ T cells, B cells, monocytes, macrophages, dendritic cells, and natural killer cells. By targeting several immune checkpoint inhibitors or blockers (e.g., PD-1, PD-L1, PD-L2, CTLA-4, LAG3, and TIM-3) expressed on the surface of immune cells, several monoclonal antibodies and polyclonal antibodies have been developed and already translated clinically. In addition, natural killer cell-based, dendritic cell-based, and CAR T cell therapies have been also shown to be promising and effective immunotherapeutic approaches. In particular, CAR T cell therapy has benefited from advancements in CRISPR-Cas9 genome editing technology, allowing the generation of several modified CAR T cells with enhanced antitumor immunity. However, the emerging SARS-CoV-2 infection could hijack a patient’s immune system by releasing pro-inflammatory interleukins and cytokines such as IL-1β, IL-2, IL-6, and IL-10, and IFN-γ and TNF-α, respectively, which can further promote neutrophil extravasation and the vasodilation of blood vessels. Despite the significant development of advanced immunotherapeutic technologies, after a certain period of treatment, cancer relapses due to the development of resistance to immunotherapy. Resistance may be primary (where tumor cells do not respond to the treatment), or secondary or acquired immune resistance (where tumor cells develop resistance gradually to ICIs therapy). In this context, this review aims to address the existing immunotherapeutic technologies against cancer and the resistance mechanisms against immunotherapeutic drugs, and explain the impact of COVID-19 on cancer treatment. In addition, we will discuss what will be the future implementation of these strategies against cancer drug resistance. Finally, we will emphasize the practical steps to lay the groundwork for enlightened policy for intervention and resource allocation to care for cancer patients.

## 1. Introduction

**Figure 1:**
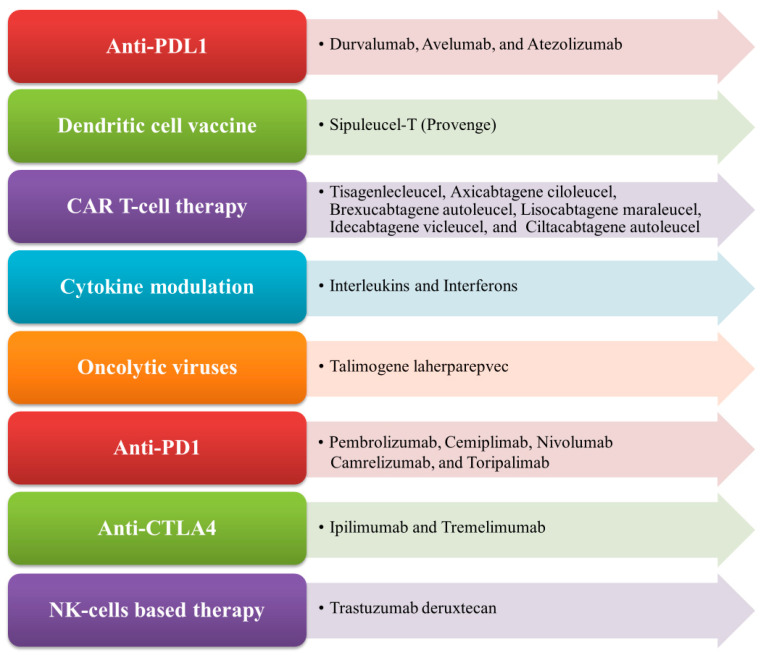
List of FDA-approved immunotherapies and their mechanism of action.

Immunotherapy is an important therapeutic strategy where extrinsic therapeutic substances either stimulate or repress the immune system to combat tumors, infections, as well as other disease ailments [1]. Immunotherapy has transpired to be the touchstone in the treatment of cancer, unlike other traditional treatment options such as targeted therapy, radiation therapy, and chemotherapy, which are emerging dynamic fields in the biopharmaceutical industry [2,3]. Cancer immunotherapy is a promising and advantageous tool for tumor treatment which acts by boosting the immune system to generate antitumor effects against tumor cells [4]. Nowadays, it gains more traction than other cancer treatments due to its ability to enhance patients’ overall survival as well as their quality of life [5]. The mechanism of antitumor immunity involves the presentation of tumor-specific antigens (TSAs) and tumor-associated antigens (TAAs) by antigen-presenting cells (APCs) such as dendritic cells and macrophages to cytotoxic T lymphocytes (CTLs) and helper T cells, thereby exerting its antitumor effects [6]. There exist various types of immunotherapy approaches in treating cancer of any origin through T-cell transfer therapy, monoclonal antibodies (mAbs), natural killer (NK) cell therapy, dendritic cell (DC)-based vaccines, gene-editing using clustered regularly interspaced short palindromic repeats-CRISPR- and its associated protein-9 (CRISPR-Cas9) technology, immune checkpoint inhibitors, as well as other cancer vaccines [7,8]. In the case of advanced tumors of various origins, the accepted treatment regime employs immune checkpoint inhibitors (ICPs or ICIs), which are well known to target programmed cell death protein 1 (PD-1)/programmed cell death-ligand 1 (PD-L1) and cytotoxic T-lymphocyte associated antigen 4 (CTLA-4) [9]. More recently, in 2020, globally active chimeric antigen receptor (CAR) T cell therapies were clinically employed to treat patients, which also encompassed advanced precision medicine immunotherapy measures like the use of checkpoint inhibitors [10,11]. The mechanism of CAR T cells involves the adoptive transfer of modified T cells, i.e., chimeric antigen receptor (CAR) T cells intended towards the cluster of differentiation 19 (CD19), which is currently authorized to cure patients with advanced B cell lymphoma and refractory B cell acute lymphoblastic leukemia-ALL [12]. In cancer immunotherapy, the DC-based therapy is specifically used to treat prostate cancer. The NK-cell-based therapy involves the modification of NK cells and enables the clinical treatment of different cancer types like breast cancer, ALL, neuroblastoma, gastrointestinal (GI)-tract cancer, etc. [13]. Nowadays, CRISPR Cas-9 technology is widely used to inactivate oncogenes by editing tumor genes with the Cas-9 endonuclease (RNA-guided mechanism) [14]. Some of the FDA-approved immunotherapies for the treatment of different types of cancer conditions, along with their mechanisms, are illustrated in Figure 1.

Despite the progress in cancer immunotherapy, there is a challenge as cancer cells can develop resistance either initially or in response to subsequent treatments, including primary, secondary, or acquired resistance. The drawback of immunotherapy resistance is more intricate because tumors subsist in a progressive microenvironment [15]. The dynamic microenvironment of tumors encompasses different types of malignant cancer cells, innate and adaptive immune components, extracellular matrix, signaling molecules, and blood vessels that act separately as well as in combination to increase the sensitivity and efficacy of immunotherapy [16,17]. In this fashion, multiple stratagems are adopted to transform the niche with the aim of improving their response to immunotherapy against cancer. These are widely classified into two types, namely, direct and indirect modulation of tumor immunogenicity. Direct modulation involves the alteration of the tumor by itself, whereas the indirect modulation of immunogenicity acts on the tumor niche. This review aims to discuss the existing immunotherapies, different types of resistance mechanisms to immunotherapy, the impact of COVID-19 on immunotherapy, future perspectives in the field, and policy recommendations for existing immunotherapies.

## 2. Modulation of Tumor Immunogenicity

Immunogenicity is the ability of a substance to provoke a protective immune response. Most preferable is eliciting the adaptive immune response of the host immune system. The immunogenicity of an antigen can be determined by the following three aspects: immunological defense (the capability to eliminate the antigens and combat pathogenic infection), immunological homeostasis (maintaining a stable homeostasis upon the recognition and elimination of damaged cells or tissue), and immunological surveillance (the capacity to recognize and kill the mutated, abnormally behaving cells, and prevent malignant growth in the body) [18].

Tumor immunogenicity is basically nothing but a tumor antigen, which elicits tumor immune response to restrict tumor growth. Several modulators have already been discovered to modify the tumor microenvironment to generate an antitumor immune response.

### 2.1. Direct Modulation of Tumor Immunogenicity

The direct modulation of immunogenicity against tumors involves radiation, chemotherapy, targeted therapy, and metabolic modifiers [19].

In chemotherapy, several cytotoxic drugs, besides their direct killing of cancer cells and preventing the cancer burden, have been used in combination with immunotherapy due to their immunomodulatory role. In destroying tumor cells, cytotoxic drugs elicit a potent antitumor immune response by releasing myeloid-derived tumor-associated antigens. Additionally, chemotherapy can reduce immunosuppressive cells such as regulatory T cells (T_reg_) and myeloid-derived suppressor cells (MDSCs). Gemcitabine (a deoxycytidine analog) and Cyclophosphamide (an alkylating agent) are examples of widely used chemotherapeutic drugs with immunotherapeutic drugs due to cross-priming and antigen cross-presentation; mechanistically, this occurs because of the suppression of T_reg_ cells, and the stimulation of the proliferation of T_eff_ cells, respectively [20]. The FDA approved the first chemotherapy combination including ICI in 2018. Carboplatin, Pemetrexed, and Pembrolizumab are now regularly used as first-line treatments for non-small-cell lung cancer (NSCLC) [21].

Radiation therapy, similar to chemotherapy, causes the direct killing of tumor cells; it also releases tumor antigens to provoke immune response, stimulate antigen presentation, and induce the infiltration of tumor-infiltrating lymphocytes (TILs) via inflammation. The innate immune systems recognize radiation-mediated DNA damage and induce the migration of T_eff_ cells to the tumor microenvironment [22].

Targeted therapy, like chemotherapy, can induce cytoreduction; additionally, it modulates immune cells, promotes the infiltration of T cells and NK cells, alters the tumor endothelium, and reduces tolerogenic cell infiltration (a heterogeneous pool of dendritic cells capable of producing immunological tolerance) [23]. The benefits of targeted therapy are that it improves the defensive ability of the immune system to fight against various types of tumors, inhibits the growth of tumors, provides inhibitory signals to the angiogenesis process of tumor cells, and initiates apoptosis or inhibits metastasis, ultimately leading to cancer cell death. Targeted therapy in the form of mAb targets specific overexpressed antigens on tumors and it can be administered intravenously [24]. The main mechanism of the induction of tumor cell death in targeted therapies is the blocking of growth factor receptor signaling. mAbs play a very significant role in blocking target receptors expressed on cancer cells. Studies have revealed that EGFR- and HER2-targeting monoclonal antibody Cetuximab causes the induction of the apoptosis of tumor cells by blocking the binding of the ligand to the particular receptor, thereby inhibiting the receptor dimerization [25,26,27].

The combined use of BRAF and MEK inhibitors against melanoma has been found to upregulate MHC levels and melanoma differentiation antigens, such as gp-100 and melanoma-associated antigen recognized by T cells (MART-1) [28]. Axitinib (vascular endothelial growth factor receptor-VEGFR-kinase) and Pembrolizumab (a humanized anti-PD-L1 mAb) have shown improved overall survival (OS) and progression-free survival (PFS) in advanced renal cell carcinoma patients [29]. Similarly, Lenvatinib (a VEGFR kinase inhibitor) in combination with Pembrolizumab was also shown to improve the health of patients with advanced endometrial cancer [30]. Combination therapy is becoming the norm in cancer treatment. Pembrolizumab and Lenvatinib were first approved by the FDA in 2014 and 2015, respectively. The combination has shown significant overall survival among patients with advanced endometrial cancer in a phase III clinical trial. The FDA also granted accelerated drug approval for Pembrolizumab for advanced endometrial cancer as a single treatment in March 2022 [31].

The intake and digestion of nutrients, as well as the removal of cellular waste, are critical aspects of every tumor’s interaction with its local milieu. Many of the substances that fuel tumor cells are also important to immune cells, complicating efforts to target metabolic pathways therapeutically. Glucose, lactate, fatty acids, and amino acids are the most significant target molecules of the cancer cells. Several therapeutic aspects have demonstrated in vitro success in modulating the tumor’s interaction with microenvironmental glucose, such as reducing glucose levels, modifying glycolytic pathways, and/or altering lactic acid metabolism [32]. Metformin and Phenformin are two important anti-diabetic drugs that have been widely used against breast cancer [33]. In a preclinical-level study, Phenformin has shown antitumor efficacy against hematological cancer and solid tumors [34]. Phenformin drugs suppress the MDSCs and enhance the ICPs in inhibitory-efficacy melanoma models [35].

### 2.2. Indirect Modulation of Tumor Immunogenicity

The indirect modulation of immunogenicity involves markers of the immunogenic microenvironment, ICPs blockers, molecules that stimulate T cells response to the tumor microenvironment, compounds that trigger stimulatory pathways, molecular vaccines (i.e., cell-based vaccines, protein/peptide vaccines, and genetic vaccines), oncolytic viruses, and epigenetic modifications [19].

Three main kinds of T cell therapy modulate the T cell response to tumor microenvironment including TILs infusion/treatment [36], CAR T cell therapy, and T-cell receptor (TCR)-engineered cell therapy. Immunogenic markers of the tumor niche are effector CD8^+^ T cells (T_eff_), regulatory T cells (T_reg_), and myeloid-derived suppressor cells. In many types of tumors, the T_eff_:T_reg_ ratio has been used as an important prognostic and predictive marker [37,38,39,40]. Additionally, tumor intrinsic factors that include PD-L1 expression and tumor mutation burden (TMB), and mismatch repair deficiency have emerged as potential biomarkers with mixed success, as clinical predictors of multiple types of cancer in response to ICP inhibitor therapy. Immunotherapies targeting PD-1/PD-L1 have yielded a noticeable clinical response against a subset of patients with melanoma, NSCLC, and urothelial cancer [41,42,43]. ICI therapy targeting TMB within the tumor genome has also shown a remarkable clinical response against multiple cancers such as NSCLC [44,45], small-cell lung cancer (SCLC) [46], melanoma [47], and colorectal cancer [48].

Activating stimulatory pathways may involve Theralizumab (TGN1412), a mAb agonist of CD28 known to interact with B7, which is a co-stimulatory molecule that activates T cells; hence, proliferated and differentiated T cells improve the antitumor immune response [49]. Moreover, inducible T-cell co-stimulator (ICOS, i.e., CD28 superfamily), Toll-like receptors (TLRs, i.e., CD40), and OX40 receptor (CD134) are some of the secondary co-stimulatory immune checkpoint targets [50]. Vaccines like cell-based vaccines, protein/peptide-based vaccines, and genetic vaccines (DNA/RNA-based vaccines) regulate the immune niche and are known to amplify the immune reaction by giving direct antigen injection or else through the lysis of tumor cells to render intratumoral immunogen. The first FDA-approved cell-based vaccine was Sipuleucel–T, which is a DC vaccine [51]. In 2018, Pan, RY et al. illustrated the power of personalized cancer vaccines mainly by concentrating on neoantigens/tumor-specific antigens, which are fabricated by non-synonymous mutation or errors in transcription in tumor cells. By utilizing next-generation sequencing, neoantigens can be characterized by evaluating cancer cells from peripheral blood mononuclear cells since neoantigens are restricted to cancer cells [52]. Tumor-specific epitopes were therefore used to assess the antigenic landscape of tumors, which allowed researchers to study the relationship between immunogenic peptides binding to the major histocompatibility complex (MHC) molecule and the presentation on the cancer cell surface to engage T cells [53,54]. Since not all neoantigens are immunogenic and due to the high degree of polymorphism in TCRs, studies have focused on developing bioinformatic tools to predict the binding ability of neoantigens to TCRs [55]. Intracellular short ~9-mer peptides bind to a protein complex consisting of β_2_-microglobulin (β_2_M) and human leukocyte antigen (HLA) proteins (HLA-A, HLA-B, and HLA-C) that are presented on the cell surface to engage CD8^+^ T cells [56]. Several processes were reported as impairing MHC function, such as somatic mutations affecting the MHC complex and its dysregulation [57]. In this process, the integrity of the MHC complex holds excellent importance for immune surveillance in relieving the potential of neoantigens to induce immune response [58,59,60]. Later, we discuss several existing immunotherapies that play a crucial role in modulating tumor immunogenicity.

### 2.3. Existing Immunotherapies for the Indirect Modulation of Immunogenicity

Fundamentally, immunotherapy is a type of cancer treatment strategy that works by boosting the immune system against cancer cells with the help of modulators (body-synthesized molecules) or biologics made in the laboratory [61]. Different immunotherapy approaches have been advanced, including the administration of therapeutic vaccines or exogenous cytokines to enhance the T cell number against tumor cells, the adoptive transfer of immune effector cells specific to tumor antigens, and nowadays, a variety of agonists of co-stimulatory receptors and ICPs that are applied to alleviate tumor-induced immunosuppressive action [62]. Here follows a description of some existing immunotherapeutic approaches.

#### 2.3.1. Checkpoint Blockade Therapy

**Figure 2:**
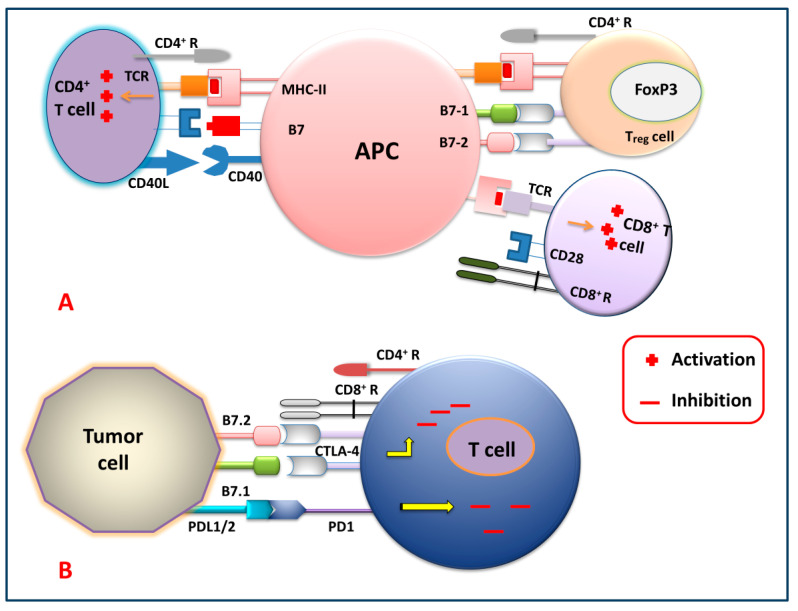
Immune checkpoint signaling pathways. (**A**) T cell (CD4^+^, T_reg_ and CD8^+^) activation by APC, and (**B**) T cell inhibition by tumor cells via targeting several immune checkpoint signaling pathways.

Figure 2 shows different immune checkpoints through which the tumor cells send inhibitory signals to the T cells, thereby inhibiting the generation of the antitumor immune response against tumor cells by suppressing the activation, proliferation, and differentiation of T cells. By blocking these checkpoints, the cancer cells cannot express their receptor against killer T cells. So, generations of these checkpoint-specific antibodies are commonly used for the signal blockade. For that reason, checkpoint-specific antibodies that are administered to the patients eventually block the cancer cells and mediate the killer T cells to target and degrade the cancerous cells precisely.

**Figure 3:**
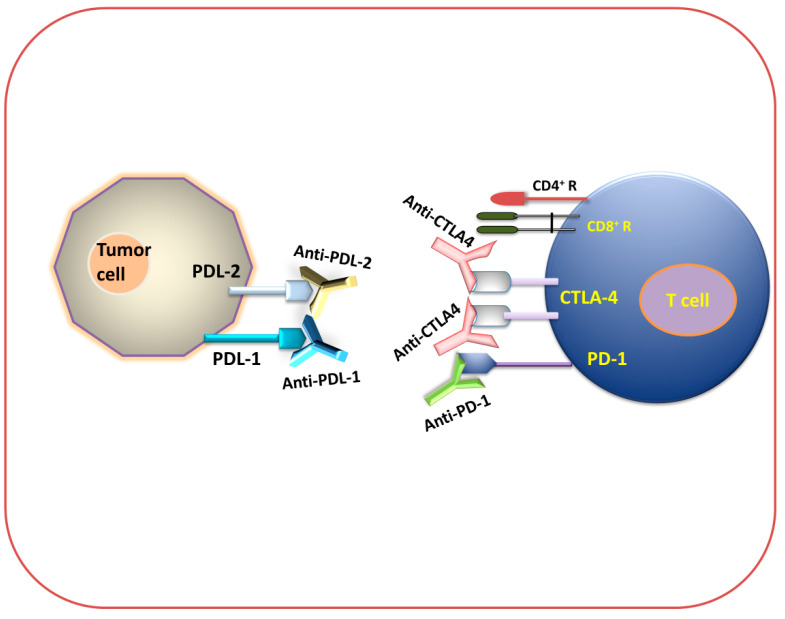
Immune checkpoint inhibition (antibody-mediated immunotherapeutic strategy). Diagram for anti-PDL-1, anti-PDL-2, anti-PD-1, and anti-CTLA-4 immunotherapy.

**Figure 4:**
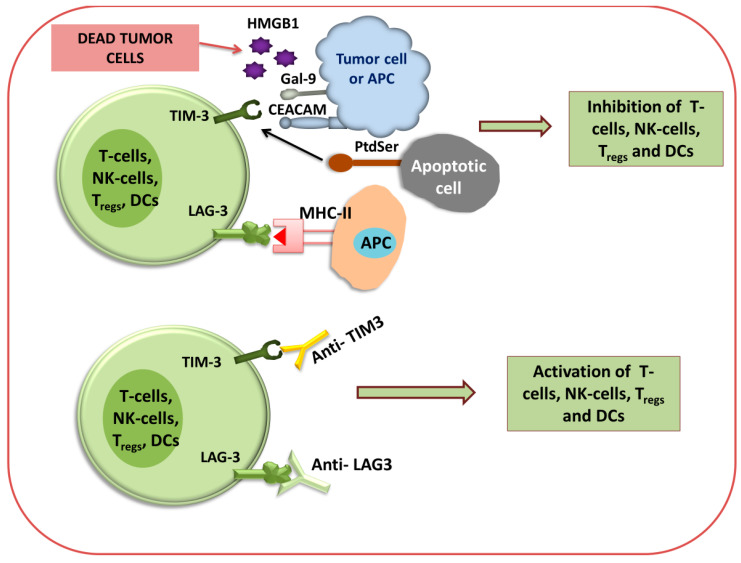
Immune checkpoint inhibition of TIM3 and LAG3 using antibodies. Diagram for anti-TIM3 and anti-LAG3 immunotherapy via the activation or inhibition of T cells, NK cells, T-reg cells, and DCs.

Figure 3 and Figure 4 show that different immune checkpoints are inhibited by different antibodies such as anti-PDL1, anti-PDL2, anti-PD1, anti-CTLA4, anti-lymphocyte-activation gene 3 (anti-LAG3), and anti-T cell immunoglobulin and mucin domain-containing protein 3 (anti-TIM-3), which are involved in the generation of an antitumor immune response against cancerous cells.

Among various types of immune checkpoints, two receptor classes are actively expressed on activated T cells, CTLA-4 and PD-1, which are targeted by cancer cells to negatively regulate the T cells’ function from killing the tumors [63]. mAb-based therapies have been used against these immune checkpoints, where they have produced a long-term durable immune response against different types of malignancies [64,65,66,67]. Furthermore, in mAb-based therapies, a specific mAb has been designed that particularly targets either CTLA-4 (such as Ipilimumab and Tremelimumab) or PD-1 (such as Nivolumab and Pembrolizumab), resulting in significant clinical advantages, including a durable immune response against different malignancies [68,69,70].

##### Anti-PD-1 Therapy 

PD-1 (CD279) is a type-I transmembrane cell surface receptor. It belongs to the B7/CD28 family. It is a negative co-stimulatory receptor, which negatively regulates T cell functions, reduces the production of cytokines, and inhibits cytolytic functions upon interaction with its ligands PD-L1 (also known as B7-H1 or CD274) and PD-L2 (also known as B7-DC or CD273) [71]. PD-1 is found on various types of immune cells, such as activated T lymphocytes, B lymphocytes, NK cells, and many subtypes of dendritic cells (DCs) and monocytes [72] that are involved in the inhibition of immune response and the promotion of self-tolerance through the modulation of T cell activity [71]. PD-1 is homologous to CD28 and normally binds to B7, a co-stimulatory molecule presented on APCs that are mainly involved in immune suppression and regulate an adaptive immune response [73]. PD-1 interferes with the function of potentially pathogenic self-reactive CD4^+^ T cells and CD8^+^ T cells, and restricts their activation. The PD-1 pathway plays a crucial role in regulating the initial activation of T cells, fine-tuning their fate and functions, promoting T cell tolerance, and restoring immune homeostasis [74]. It is also reported that tumor-specific T cells such as TILs express high levels of PD-1 on their surface, which can lead to a weakened antitumor immunological response [75]. PD-1 is also expressed on the surface of myeloid dendritic cells, Langerhans cells, and mast cells to regulate their cell functions in different pathophysiological conditions [76]. Tumor-infiltrating regulatory T cells (T_reg_) express a high amount of the PD-1 receptor so that it can interact with PD-L1 and PD-L2, resulting in the inhibition of the antitumor immune response [77]. Therefore, using mAb against PD-1 blocks the inhibitory signal to effector T cells, thus leading to the inhibition of immunosuppression and the enhancement of the antitumor immune response. The FDA has approved several PD-1 blockers after getting positive results in clinical trials, such as Pembrolizumab, Nivolumab, Cemiplimab, Camrelizumab, and Toripalimab (see Figure 1).

Pembrolizumab and Nivolumab are humanized IgG4-type mAbs that block the interaction of PD-L1 and PD-L2 with PD-1, thereby promoting an immune response to generate antitumor immunity [78,79]. Pembrolizumab has been approved to treat advanced melanoma, advanced PD-L1-positive NSCLC, metastatic urothelial carcinoma (UC), metastatic head and neck squamous-cell carcinoma (HNSCC), hematologic malignancy, microsatellite instability or mismatch repair-deficient cancers, advanced gastroesophageal cancer, metastatic cervical cancer, and locally advanced or metastatic esophageal squamous-cell carcinoma (ESCC) [79]. Nivolumab has been approved by the FDA to treat deficiency mismatch repair (dMMR) or MSI-H metastatic colorectal cancer, melanoma, metastatic squamous NSCLC, metastatic non-squamous NSCLC, locally advanced or metastatic UC, advanced renal cell carcinoma, hematologic malignancy, and advanced hepatocellular carcinoma [79]. The FDA-approved Cemiplimab has been used as a first-line treatment for patients with advanced NSCLC [80] and advanced cutaneous squamous-cell carcinoma (CSCC) [79]. Currently, Camrelizumab and Toripalimab have been approved by the FDA to treat classical Hodgkin lymphoma (cHL) and malignant melanoma in a phase II clinical trial [79]. For detailed history approval, refer to the drugs.com website.

##### Anti-PD-L1 and PD-L2 Therapy

PD-L1 and PD-L2 are the two vital ligands of PD-1 and are important immune checkpoint targets. Antibodies targeting this checkpoint can block the interaction with PD-1 and boost immune response against tumor specific antigens [81]. PD-L1 is found on activated T cells and B cells, dendritic cells, and macrophages, as well as being expressed on APCs and cancer cells. It is widely used by tumor cells to escape from host immune response [81]. Upon recognition of PD-1 on T cells by PD-L1 on tumor cells, the PD-L1 protein level becomes unregulated on tumor cells to promote the apoptosis of PD-1-expressing T cells [82]. The inhibition of PD-L1 disrupts its binding to PD-1, thereby preventing the suppression of T cell activation and proliferation, and enhancing the long-term immune response together with antitumor immunity against a wide range of cancers [71]. Several inhibitors have been developed against PD-L1. Currently, the FDA has approved three PD-L1 inhibitors, which are Atezolizumab (MPDL3280), Durvalumab (MEDI4736), and Avelumab (MSB0010718C) (Figure 1), that are being used to treat different cancer types such as NSCLC and Merkel cell carcinoma (MCC) [83].

The antibodies Atezolizumab, Durvalumab, and Avelumab are all humanized IgG1-type mAbs that bind specifically with high affinity to PD-L1 to block the binding with both PD-1 and B7 (CD80, CD86), thereby increasing the level of proliferated CD8^+^ T cells. Atezolizumab (Figure 1) transiently increases the different cytokine levels of interferon-γ (IFN-γ), interleukin 8 (IL-8), and C-X-C motif chemokine ligand 11 (CXCL11), and it decreases the levels of IL-6 along with the induction of other cytokine changes [84,85]. Durvalumab (Imfinzi) (Figure 1) is the most potent ICP inhibitor drug and is being used to treat certain types of malignancies such as bladder-urothelial cancer (UC), lung cancer (NSCLC and SCLC), and biliary tract cancer (BTC) [86,87]. A Durvalumab and Tremelimumab (Figure 1) combination therapy has shown clinical benefits against solid tumors such as HNSCC [88] and unresectable hepatocellular carcinoma (uHCC) [89]. This combination is also being evaluated in phase II and phase III clinical trials for various cancers such as metastatic gastric cancer (mGC) [90] and mNSCLC [91], and has been shown to be very effective in the treatment of these cancers. This compound is designed to prevent antibody-dependent cell-mediated cytotoxicity (ADCC) on activated T cells expressing PD-L1 [92,93]. Avelumab (Figure 1) also blocks the interaction of PD-L1 with inhibitory T-cell receptor PD-1 and co-stimulatory molecule B7.1 [94]. Collectively, all these drugs reduce the immunosuppressive signals by increasing T cell-mediated immunity within the tumor microenvironment (TME), paving the way for anticancer therapy.

PD-L2 binds to its appropriate receptor PD-1 with a 2-to-6-fold higher affinity when compared to PD-L1 [95]. PD-L2 is expressed on the surface of different types of APCs such as macrophages, mast cells, DCs, certain B lymphocyte cell populations, and intestinal stromal cells; additionally, it plays a role in immune tolerance [96,97]. Granulocyte-monocyte colony-stimulating factor (GM-CSF) and IL-4 cause the induced expression of PD-L2 on DCs, and thus promote immune-independent tumor progression [98]. GM-CSF has been found upregulated in various malignancies such as skin cancer, gliomas, HNSCC, and lung cancer [99], and IL-4 has also been found upregulated in several epithelial cancers such as breast, pancreas, prostate, and colon cancer [100]. Various studies reported that the blockade of PD-1 and PD-L2 interaction using mAb enhanced antitumor immune response [101]. Tumor cells not only can upregulate the level of GM-CSF and IL-4, but they also express PD-L2 on their surface for immune suppression [102,103]. Preclinical studies of lung squamous-cell carcinoma and renal cell carcinoma reported that cancer cells with PD-L2 expression on their surface could effectively inhibit CD8^+^ T cell functions and play an important role as pro-tumor cells in the TME. This might be overcome by using combined mAbs against PDL-2 ICPs [104].

##### Anti-CTLA-4 Therapy

CTLA-4 receptor is expressed on both CD4^+^ helper T cells and CD8^+^ cytotoxic T cells to negatively regulate T cell activation [105]. It falls under the superfamily of CD38-B7 [106]. Two distinct ligands of CTLA-4 are CD80 and CD86, which are also known as B7.1 and B7.2, respectively. CTLA-4 negatively regulates T_reg_ cell activation against tumor-associated antigens (TAAs) by interacting with their ligands, B7.1 and B7.2, with higher affinity compared to CD28 binding to these ligands, and downregulates immune response against tumor [107]. Through this mechanism, CTLA-4 interferes with the co-stimulatory signaling cascade initiated by the interaction of CD28 with the B7 molecule on the APCs. Generally, CD-28 transduces the activating and proliferating signal to T cells via Ak strain transforming (AKT), a serine/threonine-protein kinase, and phosphoinositide 3-kinase (PI3K pathway) [108]. Studies have reported that the use of mAbs against this immune checkpoint has shown a remarkably potent induction of T cell activation and an increase in the number of tumor-specific T_reg_ cells in recognition of tumor immunogen to elicit an antitumor response [109,110,111]. Cancer immunotherapy clinical trials reported that mAbs against CTLA-4 have established unprecedented therapeutic advantages and generated a long-term durable immune response in multiple types of advanced cancers [66,67,112].

The FDA has approved the two human mAbs Ipilimumab and Tremelimumab (Figure 1), which bind to CTLA-4 specifically, thereby blocking their interaction with B7.1 and B7.2, leading to antitumor immune response induction against various types of tumor cells. It has also been reported that the administration of anti-CTLA-4 in patients with metastatic melanoma has shown a durable tumor immune response in phase I and phase II clinical studies through CD28–B7-mediated T_reg_ cell activation against TAA [113,114].

##### LAG3 and TIM-3 Immunotherapy

Besides the above-mentioned immunotherapies, other well-known immune checkpoint inhibitors have recently emerged, such as lymphocyte activation gene 3 (LAG3), also known as CD223 [115], and T-cell immunoglobulin and mucin domain 3 (TIM-3) [116]. Several studies have reported that in the tumor microenvironment, LAG3 is expressed on the surface of TILs (CD4^+^ and CD8^+^ T cells), including T_reg_ cells, B cells, NK cells, NKT cells, plasmacytoid DCs (pDCs), and tumor-associated macrophages (TAMs) [117,118].

It has been illustrated that the binding of LAG3 to the primary ligand MHC class II results in the repression of effector T cells like PD-1 and increases the regulatory T cell activity, thereby supporting tumor growth [119]. By binding to the MHC-II molecule, LAG3 negatively regulates the CD4^+^ T cells’ and CD8^+^ T cells’ functions and their response. TIM-3 is constantly expressed on DC cells, particularly on DC1 cells in tumors. Also, it facilitates cross-presentation of the antigen and licenses CD8^+^ T cells [120,121]. Most terminally dysfunctional subsets of CD8^+^ TILs are marked by TIM-3 [122]. Clinical trials demonstrated that anti-LAG3 antibody administration can enhance immune response [123]. Co-inhibitory receptor TIM-3 expressed on IFN-γ-secreting CD4^+^ T cells and CD8^+^ T cells, T_reg_ cells, NK cells, and monocytes. TIM-3 plays an important role in the regulation of type-1 T-helper cell (Th-1)-mediated immune response, and also regulates the signaling of various cytokines (TNF-α and IFN-γ) plus their release from T cells [123]. Some studies reported that in renal cell carcinoma, a higher expression of TIM-3 was also detected on CD204^+^ TAMs and tumor cells [124]. Four different ligands that bind to TIM-3 are galectin-9, phosphatidylserine (PtdSer), high-mobility group protein B1 (HMGB1), and carcinoembryonic antigen-related cell adhesion molecule-1 (CEACAM) [125]. As TIM-3 has a negative impact on immune regulation, the blockade of TIM-3 inhibition using an antibody may induce an antitumor immune response against some cancers, such as colorectal cancer, in which anti-CTLA-4 and anti-PD-1 antibodies were not effective [126].

#### 2.3.2. CAR T Cell Therapy

**Figure 5:**
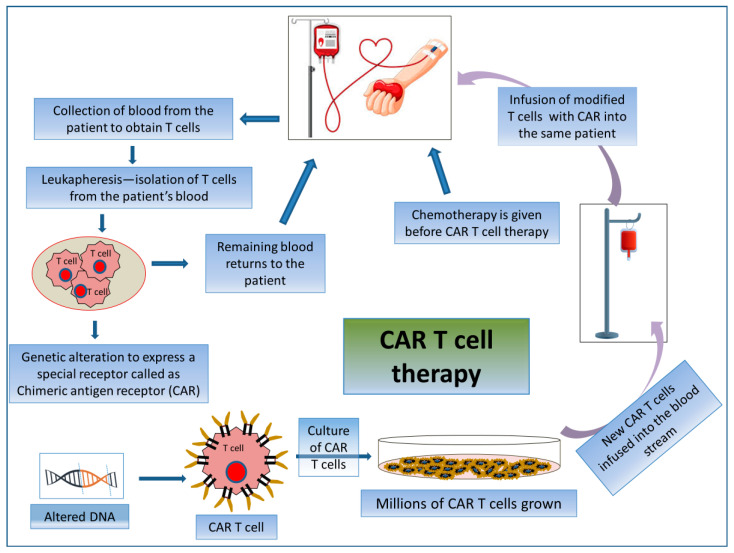
Mechanism of action involved in CAR T cell therapy by acquiring patient’s own T cells. Schematic representation of the process of CAR T cell therapy.

CAR T cell therapy is an adoptive T cell transfer (ACT) therapy whereby killing cancerous cells does not involve MHC molecules but rather the patient’s T-lymphocytes. CAR T cell therapy encompasses the exogenous selection, modification, expansion, and re-infusion of modified T cells into the patient’s body (Figure 5). CARs are generated by collecting patient T cells via a process known as apheresis, and engineered cells are reintroduced after a preparative regimen. Here, T-lymphocytes are collected from their peripheral blood and then are genetically modified ex vivo via the single-chain variable fragment (scFv) antigen recognition domain to express CD19-specific CARs. Thus, the reinfused modified T cells seek out and destroy the tumor cells expressing CD19 in the patient. By this mechanism, modified T cells raise an immunogenic niche inside the patient’s body [127]. These are used specifically for treating patients with advanced-stage B-cell malignancies [128].

First-generation CAR T cells bind to a receptor derived from an extracellular antibody mainly designed for a tumor antigen with a CD3-based intracellular activating domain [129]. Second- and third-generation CAR T cells are formed based on different molecular components of co-stimulatory molecules, including CD28, ICOS, OX40 (CD134), and 4-1BB (CD137), to generate durable immune response even after repeated times of antigenic stimulation [130,131,132]. Fourth-generation CAR T cells contain signaling domains from an inducible expression of different inflammatory cytokines, e.g., IL-12 or IL-18, or cytokine receptors [133,134]. The most successful FDA-approved CAR T cell therapy, Tisagenlecleucel, Brexucabtagene autoleucel, and Axicabtagene ciloleucel (Figure 1), targets the B-cell lineage antigen CD19. The CD-19-targeted CAR T cells have been used as an important option to treat patients with certain incursive B-cell non-Hodgkin lymphomas (NHLs) and/or acute B-cell lymphoblastic leukemia (B-ALL) [130]. Recently, the FDA approved several CAR T cell therapeutic regimens, such as Lisocabtagene maraleucel, which targets CD19 antigen and is used to treat relapsed or refractory large B-cell lymphoma; and Idecabtagene vicleucel and Ciltacabtagene autoleucel both of which target B cell maturation antigen (BCMA) and are used against relapsed or refractory multiple myeloma [135].

#### 2.3.3. Natural Killer (NK) Cell-Based Immunotherapy (Alternative to CAR T Cell Therapy)

NK cell immunotherapy for cancer has been extensively reviewed, presenting a perspective on NK cell biology and function, therapy types, and clinical trials [136,137]. NK cells play a very specialized role in innate immune defense and have a potent activity in the killing of abnormal cells (virus-infected or cancer cells) in an MHC-unrestricted manner [138,139]. NK cells have the ability to recognize metastatic cells and kill these cells by releasing either lysing enzymes or through ADCC. For that, NK cells do not need any prior sensitization of any particular antigen. Upon activation in the presence of malignant cells, NK cells mimic the activated cytotoxic T cell activity against antigens by secreting cytotoxic molecules containing perforin and granzyme granules to lyse these malignant cells directly. NK cells also have a role in modulating adaptive immunity by producing cytokines and chemokines such as TNF-α and IFN-γ. For many decades, NK cells have been investigated to be used against cancer [138]. The efficacy of NK cells can be increased by using some immune stimulants, particularly various types of antibodies and cytokines during antitumor immunotherapy [140] including adoptive transfer (direct transfer into the patients) of ex vivo cultured NK cells [141]. CAR NK cells are constructed by genetic modification of NK cells from different sources, including hematopoietic pluripotent stem cells (HPSCs), primary NK cells, as well as NK cell lines, to express CARs in order to enhance the recognition of a particular surface marker of cancer cells such as CD19, CD20, and ErbB2. After recognition of the target, the downstream signaling domain (such as CD3ζ and CD28) of CAR causes the activation of PI3K and ultimately influences the release of IFN-γ and stimulates cytotoxicity [142,143].

CAR NK cells have both CAR-dependent and CAR-independent potential cytotoxic effects against cancer cells [144]. Some preclinical studies suggested that NK-cell-derived extracellular vesicles (EVs) have potent antitumor activity. NK EVs bear the CD56 marker and contain some lytic proteins like perforin-granzyme and FasL [145]. Other studies have reported that NK EVs showed potent cytotoxic effects of killing against various cancer cell lines together with breast carcinoma, ALL, and neuroblastomas [145]. In human epidermal growth factor receptor 2 (HER2)-positive breast cancer patients, Trastuzumab deruxtecan (see Figure 1) was used as a monotherapy and was found to stimulate the activation of NK cells against HER2. Dual HER2 blockage with Trastuzumab and Pertuzumab is currently the mainstay of therapy in early and advanced cancer illness, since this combination may have an additive impact on ADCC [146].

#### 2.3.4. Dendritic Cell Vaccine Therapy (a Cross between a Vaccine and a Cell Therapy)

DC vaccines elicit a specific immune response that can selectively eliminate cancer cells. Progress in this area has been recently reviewed [147]. DCs have been used in clinical trials to test their application in boosting antitumor immunity [148]. DCs are a heterogeneous population of different types of dendritic cells comprising conventional dendritic cells 1 (cDC1), conventional dendritic cells 2 (cDC2), monocyte-derived DCs (MoDCs), and pDCs developed from hematopoietic cells. They are involved in maintaining the connection between innate and adaptive immunity. DCs are used in vaccine production due to their ability to express TAAs primarily on CD4^+^ T cells, and cross-presentation (i.e., cross-priming) to CD8^+^ T cells [149,150]. As DCs are found across the skin, mucosa, blood, and as well as lymphoid tissues, they have the capacity of antigen processing and presentation to naïve T-lymphocyte cells, consequently inducing them to convert into tolerogenic subsets or effector subsets. Therefore, they can orchestrate an adaptive immune response [151,152,153]. DCs have the capacity to cross the representation of exogenous tumor antigens by class-I MHC molecules to recently matured CD8^+^ T lymphocyte cells from the thymus. In addition, they have the ability to polarize the CD4^+^ T cells towards the Th-1 subset effectively and to activate the NK cells [154,155]. Moreover, activated and proliferated CTLs trigger the process of elimination of tumor cells by recognizing the antigenic peptide complex with the MHC-I molecule presented on the tumor cells [156].

DC-based vaccines induce the activity of CTLs expressing low levels of CTLA-4 and PD-1 to increase their cytolytic ability and to enhance the expression of different molecules (CXCR3 and CD103/CD49a) to empower the migration of CTLs towards the tumor microenvironment [157]. Currently, the FDA has approved Sipuleucel-T (Figure 1) as the first DC-based autologous cellular immunotherapeutic drug to treat prostate cancer. The major component of Sipuleucel-T is the fusion protein (PA2024) composed of two constituents which are cancer antigen-prostatic acid phosphatase (PAP) conjugated to adjuvant GM-CSF. Some clinical studies have reported that under ex vivo conditions, this fusion protein (PAP-GMCSF) undergoes activation when incubated with the isolated APCs. These activated APCs can now fight against prostate cancer cells once re-infused into the patients [158,159].

#### 2.3.5. CRISPR-Cas9-Based Immunotherapy

Few reviews tackled the cutting-edge application of gene editing in cancer immunotherapy [14,160]. CRISPR-Cas9 is a genome-editing technology tool that uses mRNA nuclease to cleave genomic DNA at a target sequence of interest with efficiency, precision, and specificity. In cancer, excessive accumulation of mutations leads to the activation of different oncogenes via the gain of proto-oncogene function and the inactivation of tumor suppressor genes via the loss of gene function. Hence, CRISPR-Cas9 can be used as an important therapeutic tool for oncogene inactivation by tumor genome editing and the restoration of tumor suppressor and apoptotic functions [161]. The CRISPR-Cas9 system has two essential components, single guide RNA (sgRNA) and Cas-9 endonuclease, which cut the site-specific double-stranded DNA with the help of sgRNA [162]. Recently, the CRISPR-Cas9 system has emerged as a therapeutic technology for generating CAR T cells in the cancer immunotherapy field. In 2017, one study reported that the CRISPR-Cas9 system could disrupt several genomic sites simultaneously to make universal CAR T cells, which have defective TCR and MHC-I expression, showing potent antitumor activity [163]. The Fas receptor, known as CD95, causes the apoptosis of expanded T cells and can damage CAR T cell functions when it interacts with the Fas ligand (FasL). Thus, using CRIPSR-Cas9 technology, Fas knockout CAR T cells can be formed, which have a better ability to eliminate tumor cells [164]. Apart from constructing universal CAR T cells, CRISPR-Cas9-mediated genome editing eliminates multiple genes which encode inhibitory T-cell surface receptor PD-1 and CTLA-4 to improve the efficacy of T cell-based antitumor immunotherapy [165].

## 3. COVID-19 and Immunotherapy

COVID-19 has been a global threat and a serious pandemic. It is an acute respiratory syndrome elicited by SARS-CoV-2. SARS-CoV-2 has a great impact on the immune dysregulation of the affected individual, surprisingly enhancing the serum levels of C-reactive protein (CRP) and IL-6, and reducing CD4^+^ plus CD8^+^ T-lymphocyte populations in the affected patients. Elevated levels of other inflammatory cytokines and chemokines like IL-2 and IL-8, with increased levels of eosinophil and neutrophils, might provoke immune abnormality in patients affected with COVID-19 [166]. COVID-19 infection exacerbates risk and mortality in cancer patients. Cancer patients are more susceptible to SARS-CoV-2 infection, which induces cancer metastatic processes via the induction of inflammation, cytokine-induced vasodilation, neutrophil extravasation, and the leaking of plasma in the affected tissue [167,168].

In COVID-19 patients, a variable immune response, including severe to moderate systemic inflammation and noticeable immune suppression, was observed [169]. It is reported that upon SARS-CoV-2 infection, the inflammasome becomes active, which was found to be associated with the severity of COVID-19 patients [170], and among them, the NLRP3 inflammasome becomes more active and can promote a cytokine storm [171]. A significant change in cytokine (including IFN-α, β, and γ; IL-2, IL-6, IL-7, IL-12, and IL-1β) and chemokine (including CCL 2, 3, and 5; CXCL10, CXCL9, and CXCL8) levels in severe COVID-19 cases was also found [172]. These exacerbated secretions of cytokines and chemokines may lead to a cytokine storm, which can further result in the development of acute respiratory distress syndrome (ARDS), respiratory failure, and organ failure, ultimately leading to death [173].

Several studies have been performed during the COVID-19 pandemic to find the impact of COVID-19 on patients receiving ICIs as immunotherapeutic treatments. The findings suggested no increase in patients’ death or disease severity nor an association between ICIs and COVID-19 disease severity in patients receiving ICIs who developed COVID-19, while other researchers concluded that “[ICPs] *not only can be safely administered to cancer patients, but they might even be beneficial in COVID-19-positive cancer patients, by exerting an immune-stimulating action*” [174,175,176]. Despite this evidence, Robilotti et al. found that immediately upon the diagnosis of COVID-19, the use of ICI was the leading risk factor for disease severity [177]. It is also reported that patients receiving immunosuppressive therapy are more susceptible to being infected by SARS-CoV-2; moreover, therapies that deplete the B-lymphocytes and reduce the production of antibodies are also a leading risk factor for COVID-19 outcome [178]. One literature review based on a case study documented that lymphoma and hematopoietic stem cell transplant patients receiving CAR T cell therapy or Rituximab treatment are susceptible to chronic infection with SARS-CoV-2 [179,180,181,182,183,184]. These cases are significant for the appearance of SARS-CoV-2 variants, which has resulted in rapid and significant mutational changes that may be responsible for novel variants of concern [185]. A systematic and meta-analysis study reported the comparable efficacy of the COVID-19 vaccine to that of cancer patients treated with ICI, and a higher efficacy than that of chemotherapy-treated cancer patients, which was measured using the seroconversion method. Furthermore, the researchers reported that the COVID-19 vaccination appears to be both safe and efficacious in cancer patients receiving ICI, although more evidence is required to further establish the robustness of these findings [186].

## 4. Resistance to Immunotherapy

Nowadays, immunotherapy has become the most efficient treatment option for a wide range of cancer types. With a prominent and long-lasting clinical response, immunotherapies provide a novel breakthrough treatment for a range of resistant carcinomas, progressively changing the pattern of tumor treatment. Despite obtaining a significant antitumor immune response after the application of CAR T cell therapy, ICI therapy (such as CTLA-4, PD-1, PD-L1, and PD-L2), NK-cell-based therapy, and the DC vaccine in a wide range of cancers, many individuals continue to have difficulty responding to cancer therapies. Various intrinsic and extrinsic mechanisms are involved in suppressing the antitumor immune response of immunotherapy, resulting in immunotherapy resistance [187]. Intrinsic mechanisms of immunotherapy resistance involve the modification of antigen recognition, gene expression, cell signaling, and DNA damage response by tumor cells, whereas extrinsic resistance involves the development of resistance outside the cancer cells throughout the T cell activation phase [188]. Even though ICP inhibitor therapy has been promising against broad types of cancers, there is still notably limited response, especially in certain cancers such as breast and prostate, making immunotherapy challenging [189]. It is reported that due to tumor intrinsic and extrinsic factors facilitating tumor heterogeneity [190], ICP inhibitors fail to show a durable response in breast and prostate cancer patients in comparison to melanoma, NSCLC, and renal cell carcinoma [46,191,192].

Usually, immune resistance is classified into primary and acquired resistance. Primary resistance of cancer to immunotherapy, where the cancer cells do not respond to the treatment, is due to several mechanisms such as the activation of ICP pathways, genetic aberrations, or alteration in protein expression, which is associated with the induction of enhanced expression of tumor-associated antigens, blocking the migration, activation, and infiltration of T cells into the tumor milieu [189,193]. A loss-of-function mutation in JAK1/2 genes results in the impairment of PD-L1 expression induced by IFN-γ, hence leading to primary resistance against PD-1 blockade therapies [194]. As primary resistance restricts treatment efficacy, new drug discovery or combinational therapeutic strategies are required to overcome this resistance [195]. Acquired resistance is the loss of positive response to immunotherapy after a certain duration of treatment after initial success [195]. Markedly, three mechanisms are involved in the development of acquired resistance, namely, the alteration of tumor functions, the activity of the tumor microenvironment, and the enhanced expression of ICI proteins [196]. Acquired resistance develops due to the genetic alteration in tumor cells over time, which leads to the suppression of the immune response to the treatment. The increased use of ICI immunotherapy targeting helps to promote the development of acquired resistance in patients. For instance, in advanced melanoma patients, melanoma relapses for around one-fourth to one-third of patients due to the acquired resistance against the respective immunotherapy treatments [197]. Several mechanisms are involved in the development of acquired resistance to ICIs, such as the absence of immunogenic neoantigens and defects in antigen presentation; mutations in JAK1 and JAK2 and IFN-γ signaling; the activation of multiple alternate immune checkpoint inhibitors; immunosuppressive factors in the TME; epigenetic alterations; and dysbiosis in the gut microbiota [198]. Tumor neoantigens have immunogenic properties and generate a durable immune response to ICI therapy [199]. Additionally, tumors enriched in neoantigens exhibit greater levels of granzyme A and perforin mRNA, as well as tumor-infiltrating cells, which are associated with an increase in T-cell-mediated cytolytic activity [47]. Therefore, the processes underlying the decrease in immunogenic neoantigen expression during immunotherapy may result in the development of acquired resistance. Neoantigens are processed by APCs and presented as peptide MHC-I complexes to T cells for activation and proliferation. Therefore, a reduction in the transcription of MHC mRNA, genome loss, and mutations in the β_2_-microglobulin (β_2_M) genes have a deleterious impact on antigen presentation and may explain why some people develop resistance to ICI treatment [200]. Two patients with Merkel cell carcinoma whose tumors had returned following an initial response to PD-1/PD-L1 inhibitors were discovered to have a transcriptional loss of the genes encoding MHC-I, according to Paulson et al. [201]. The Janus kinases 1 and 2 (JAK1/2) signaling cascade-mediated release of IFN-γ from tumor-specific T cells induce chemokine secretion to attract immune cells for the killing of tumor cells by promoting apoptosis [194]. The deactivating mutation of the JAK1/2 signaling cascade results in the development of IFN-γ-mediated acquired resistance, which negatively impacts immune surveillance and promotes cancer cell proliferation. Numerous studies have demonstrated that the absence of JAK/STAT signaling results in the development of PD-1 and CTLA-4 inhibitor therapy due to the inability to increase the expression of PD-L1 and MHC-I [202]. The increased expression of alternative immune checkpoints such as TIM-3, LAG-3, and B and T lymphocyte attenuator (BTLA), T cell Ig and ITIM domain (TIGIT), and V-domain Ig suppressor of T cell activation (VISTA) has been found to be associated with the development of acquired resistance to ICIs. A study reported that the increased co-expression of CTLA-4, PD-1, TIM-3, LAG-3, and BTLA was found to be positively associated with increasing T cell depletion and consequent resistance to anti-PD1 treatment in NSCLC [203]. These are a few mechanisms that have been found to be involved in the development of acquired resistance to ICIs. This dynamic nature of immunotherapy resistance can be attributed to evolving cancer cells with their microenvironment and introduced interventions such as surgery, chemotherapy, targeted therapy, and radiotherapy [204].

Furthermore, it has been found that immune functions are modulated by the gut microbiome composition and nutritional status, and that features of nutritional status and gut microbiome are associated with different responses to ICP inhibitors [205]. We believe that this is a pioneering, fertile, and exciting area of research. In addition, cancer develops adaptive immune resistance, which is a newly proposed mechanism different from targeted therapy and chemoradiotherapy, representing a mechanism for cancer cells to evade the immune system and escape from the immune-recognition mechanism by altering themselves or adapting to an immune attack. This adaptation happens within the tumor cells throughout therapy and is a type of resistance that develops because of the prescribed treatment [195]. We believe that this is another vibrant area of research.

Apart from these immune resistance mechanisms to ICI therapy, other resistance mechanisms to immunotherapy also promote cancer progression by immunosuppression, such as the accumulated heterogeneous subpopulation of MDSCs. These are generally immature myeloid cells, which can suppress the function of T cells and NK cells associated with disease progression and recurrence; reduce the immune response to the immunotherapies and the endothelium of TME, once larger than normal size; gain protumorigenic properties; and promote immune evasion and immune-resistance to the immunotherapies [206]. The cancer cells can escape from T cell-mediated attack through the inhibition of T cell activation by suppressing the antigen expression and altering the tumor antigenicity via antigenic drift (mutation in the epitope region to produce different variants) [189]. It is also reported that cancer cells developed secondary resistance which is similar to acquired resistance, where the development of disease occurs after the earlier benefits of immunotherapy. Secondary resistance mostly develops against ICI therapies to overcome the continuous immune attack. The mutation of several genes is related to the immune response signaling pathways, such as the antigen processing signaling pathway and the interferon signaling pathway. Gao et al. reported that melanoma patients with a defect in the genes of the γ-interferon pathway failed to respond to ipilimumab therapy [207]. Zatersky et al. reported that the JAK1 and JAK2 mutations and the β2M mutation led to defects in interferon signaling and antigen presentation, respectively, in melanoma patients, and the same was true for the PTEN mutation in uterine leiomyosarcoma patients who initially responded to Pembrolizumab [197]. These findings are quite intriguing since they offer the type of crucial knowledge required to comprehend the processes underlying primary and secondary resistance to immunotherapies and, in turn, create a foundation for the advancement of improved immunotherapy.

## 5. Policy Recommendations Regarding Immunotherapy

**Figure 6:**
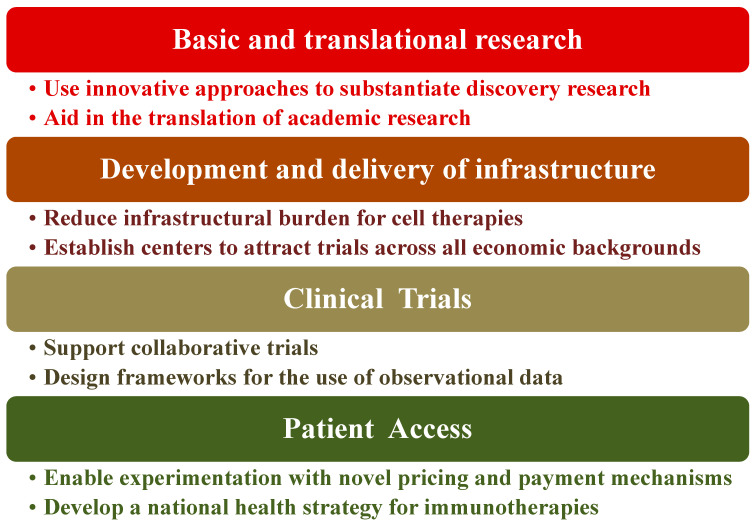
Proposed path for policy recommendation.

Immunotherapy as a treatment method for diseases is at a point of exceptional opportunity, especially for cancers. The activation or suppression of patient’s immune system has shown unparalleled clinical benefit. To maximize the impact of immunotherapy treatments, certain healthcare policies need to be in place or at the very least compose an agreed-upon framework to harness clinical advantages to patients. Indeed, there are key challenges to healthcare systems for realizing the promise of immunotherapy. The four basic elements of such challenges are shown in Figure 6 along with their potential solutions to mitigate the risks of these components [208]. We view this as an oversimplification of the problem and therefore the issues that are being faced or the solutions being proposed. Consider, for instance, the cost and resources required for low-to-medium-income countries (LMIC) even if biosimilars are available. Novel immunotherapy treatment including hospitalization can range from ca. USD 100 K to >USD 1 M/year in the US [208] along with the prerequisites for state-of-the art facilities. Overcoming these hurdles requires that governments seek strong partnerships, not only locally but also globally, with stakeholders such as the WHO, biopharma companies, and international philanthropic organizations. Furthermore, governments ought to put mechanisms in place that are outcome-based to facilitate immunotherapy access. Each healthcare system is a unique ecosystem with its own benefits, disadvantages, and implementation requirements. Hence, most likely this will be an iterative process, which requires flexibility from all engaging parties and the co-operative engagement of the regulatory authorities to create consistent frameworks and standards for best practices including information management [209,210].

The global response to and experience of the COVID-19 pandemic are instructive in this case. Therefore, we recommend the establishment of comprehensive cancer centers for excellence that meet US NIH-NCI standards, as we believe that it goes a long way towards realizing the vision and implementation of healthcare policies. Three successful examples from LMICs are from Africa, Uganda—The Uganda Cancer Institute (UCI); the Middle East, Jordan—The King Hussein Cancer Center (KHCC); and Asia, India—The Tata Memorial Hospital (TMH). Our view is that these strategies and actions would enable potentially life-changing immunotherapy treatments to reach patients sooner [209]. We understand that the features of cancer differ among various patients. Indeed, substantial genomic alterations often occur during disease progression. Appreciating genomic diversity and ethnic disparities goes a long way towards understanding tumor profile, offering therapy guidance, and enhancing the management of patients and appropriate healthcare policies; therefore, it is overall a smarter type of care, thus minimizing healthcare waste [211,212].

## 6. Future Perspectives

Although the development of several types of therapeutic strategies has significantly improved cancer treatment, the harsh reality is that the “war on cancer” is still being wagged. Even now, major challenges still exist with the currently available marketed FDA-approved drugs, including limitations associated with mAbs’ intrinsic and acquired mechanism of resistance that causes tumor heterogeneity and patient relapse [213,214]. Nowadays, the advancements of various immunotherapy-based strategies have successfully transformed the treatment of several types of tumors. The anticancer activity of the immune system has been exploited and boosted by using ICP inhibitors, the CAR modification of T cells, DC-based therapy, NK-CAR T, and CRISPR-Cas9 technology, demonstrating a successful broad spectrum of therapeutic strategies’ utility in immunotherapy to combat cancer [213,214]. Clinical research is ongoing using established therapeutic modalities and novel therapeutic approaches in immunotherapy [215]. Combining mutational burden to predict clinical response along with the development of cancer vaccines will potentially provide opportunities toward precision immunotherapy. Apart from this, urgent needs still exist in terms of discovering novel antitumor agents with higher specificity, lower toxicity, and improved efficacy [213,214,215]. Overcoming treatment limitations and/or drug resistance is a critical prerequisite to minimize patient relapse [213,214,215]. Moreover, there is a dire need for novel biomarkers across the value chain in cancer, from drug discovery and development, prevention, and diagnosis to treatment, in order to transform the way we approach cancer [209] and to truly have relevance in terms of precision medicine [212] and precision health [216].

## 7. Conclusions

The human protein repertoire plays a predominant role at the cellular level in health, disease, and therapy. Deciphering human proteins in terms of structure/function is critical to both basic and translational research including cellular therapy. This paper is intended to be comprehensive in providing a in-depth overview for the uninitiated in the field, alongside health professionals and clinicians. It presents in detail the currently used immunotherapy approaches in the treatment of cancer patients. Moreover, this review illustrates the prescience of the next frontier in the field of immunotherapy. The discussion of cutting-edge techniques focuses on both the direct and indirect modulation of tumor immunogenicity in the tumor niche in order to sensitize resistant tumors to immunotherapy. Immunotherapy plays a magnificent role in assisting patients enduring an array of cancer-associated processes. Subsequently, to limit the immunotherapy effect, cancer cells also develop resistance to the treatment. Therefore, each of the treatment options has encountered a diverse echelon of success rates along with their inimitable challenges in clinical enhancement, fabrication, delivery methods, and access to patients. One goal of this paper is to provide a proof-based resolution for governments, funders, and policymakers to organize the discovery, development, and delivery of immunotherapies globally to take full advantage of the existing capability of these transformative treatments. Since governments and policymakers can accelerate the advancement in immunotherapy by substantiating the novelty in the trial outline, the way of approaching these kinds of therapy-based treatment methods for immunotherapy in the future should purely rely on outcome-based payment for the patients rather than charging for the cost of the dosage precisely.

## Data Availability

This is literature-based review article. No new data were created or analyzed in this study. Data sharing is not applicable to this article.

## Abbreviations

CAR—chimeric antigen receptor, TSAs—tumor-specific antigens, TAAs—tumor associated antigens, mAbs—monoclonal antibodies, NK cell—natural killer cell, DC—dendritic cell, CRISPR—clustered regularly interspaced short palindromic repeats, Cas-9—CRISPR-associated protein-9, ICPs or ICIs—immune checkpoint inhibitors, PD-1—programmed cell death-protein 1, PD-L1—programmed cell death-ligand 1, CTLA-4—cytotoxic T-lymphocyte associated antigen 4, ALL—acute lymphoblastic leukemia, CD19—cluster of differentiation 19, GI—gastrointestinal, FDA—Food and Drug Administration, TIL—tumor-infiltrating lymphocytes, TCR—T-cell receptor, Treg—regulatory T cell, TMB—tumor mutation burden, NSCLC—non–small-cell lung cancer, SCLC—small-cell lung cancer, TGN1412—Theralizumab, ICOS—inducible co-stimulator, TLRs—Toll-like receptors, HLA—human leukocyte antigen, MHC—major histocompatibility complex, LAG3—Lymphocyte-activation gene 3, TIM-3—T cell immunoglobulin and mucin domain-containing protein 3, MCC—Merkel cell carcinoma, IFN-γ—interferon-γ, IL—interleukin, CXCL11—C-X-C motif chemokine ligand 11, HNSCC—head and neck squamous-cell carcinoma, ADCC—antibody-dependent cell-mediated cytotoxicity, APCs—antigen-presenting cells, GM-CSF—granulocyte-monocyte colony-stimulating factor, TME—tumor microenvironment, TGF—transforming growth factor, IgG4—immunoglobulin-G4, TAA—tumor-associated antigens, AKT—Ak strain transforming (Protein kinase B), PI3K—phosphoinositide 3-kinase, NKT cells—natural killer T cells, PtdSer—phosphatidylserine, HMGB1—high-mobility group protein B1, CEACAM—carcinoembryogic antigen-related cell adhesion molecule-1, cDC1—conventional dendritic cells 1, cDC2—conventional dendritic cells 2, MoDCs—monocyte-derived DCs, pDCs—plasmacytoid DCs, CTLs—cytotoxic T-lymphocytes, PAP—prostatic acid phosphatase, ACT—adoptive T cell transfer, scFv—single-chain variable fragments, NHLs—non-Hodgkin lymphomas, B-ALL—B-cell acute lymphoblastic leukemia, sgRNA—single guide RNA, FasL—Fas ligand, IV—intravenous, SARS-CoV-2—Severe Acute Respiratory Syndrome Coronavirus, SARS COVID-19—Severe Acute Respiratory Syndrome Coronavirus Disease-19, COVID-19—coronavirus disease-19, HPSCs—hematopoietic pluripotent stem cells, EVs—extracellular vesicles, HP—hyperprogression, MDM2—murine double minute 2, KRAS—Kirsten rat sarcoma virus, NK-CART—natural killer-chimeric antigen receptor T cells, Th1 cells—Type1 T-helper cells, TILs—tumor-infiltrating lymphocytes, MDSCs—myeloid-derived suppressor cells, BCMA—B cell maturation antigen, LMIC—low-to-medium-income countries, UCI—The Uganda Cancer Institute, KHCC—The King Hussein Cancer Center, and TMH—The Tata Memorial Hospital.
